# Structure and Function of the Extracellular Matrix in Normal and Pathological Conditions: Looking at the Bicuspid Aortic Valve

**DOI:** 10.3390/ijms262210825

**Published:** 2025-11-07

**Authors:** Francesco Nappi

**Affiliations:** Department of Cardiac Surgery, Centre Cardiologique du Nord, 93200 Saint-Denis, France; francesconappi2@gmail.com or f.nappi@ccn.fr; Tel.: +33-149334104; Fax: +33-149334119

**Keywords:** collagens, elastin, extracellular matrix, glycosaminoglycans, heparanase, hyaluronan, hyaluronidases, integrins, laminins, matrix metalloproteinases, proteoglycans, tenascins

## Abstract

This review will focus on the structure and role of the ECM in physiological conditions and pathological alterations, based on a cardiological case. The patient has a particular case of connective tissue disease (CTD), specifically bicuspid aortic valve type (BAV). The presented clinical case is as follows: a 34-year-old patient has been diagnosed with BAV. The subject is concerned about how his condition may affect his daily life. The subject is worried about passing the disease on to his children. He asked experts for advice on the causes, possible consequences and treatments. BAV is a major congenital heart defect, affecting 1–2% of the global population. This review provides an overview of the structure and function of the ECM, which plays an important role in the architecture of heart valves and vascular structures associated with connective tissue disease. The BAV has been observed to affect the connective tissue, although the underlying causes remain unclear. ECM is a 3-dimensional network of macromolecules that provides structural support for cells and tissues. Extensive research has established the regulatory functions of ECM, given its role in orchestrating cell signalling, functions, properties and morphology. Extracellular and cell-bound factors represent a substantial proportion of the major constituents of the ECM. The following proteins and glycoproteins are of particular interest: collagen, elastin, laminins, tenascins, proteoglycans, glycosaminoglycans and hyaluronan. Relevant cell receptors include CD44.

## 1. Introduction

### 1.1. General Principles on Structure and Function of the Extracellular Matrix

**Figure 1:**
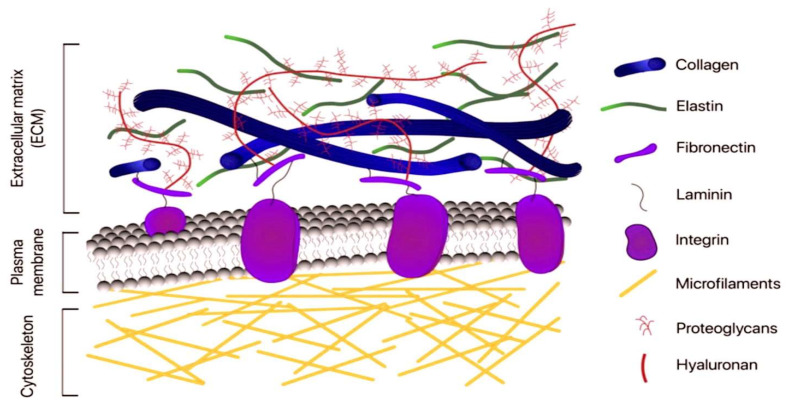
The following schematic overview illustrates the composition of the extracellular matrix and its main constituents. While the ECM is subject to variation depending on the tissue, the matrix primarily comprises a range of fibrous proteins (collagen, elastin, fibronectin, and laminin) and polysaccharides. These are produced locally and assembled into a structured network in close proximity to the cell from which they are secreted. Abbreviation; ECM, extracellular matrix From Pole et al. Cells. 12 July 2021; 10(7) Ref. [17].

The extracellular matrix is a complex, organised 3D network that plays essential roles in tissue arrangement and remodelling, and in the modulation of cellular mechanisms [1,2]. It comprises collagens, proteoglycans (PGs), glycosaminoglycans (GAGs), elastin and elastic fibres, laminins, fibronectin, and additional structural and functional macromolecules, including matricellular proteins [1,2,3,4]. ECMs communicate between cells in organs and tissues, coordinating regulatory signals [1,2,5]. They guide tissue [3]. The processes of morphogenesis, development and homeostasis are interconnected and achieved through the regulation of growth, survival, differentiation and adhesion [4]. In pathological conditions, ECMs are susceptible to remodelling, acquiring a pivotal role in the advancement of the associated pathological process [5,6,7,8,9,10,11,12]. The arrangement of different ECM types is vital for many biological functions. Tissues (epithelial, nervous, muscle and connective) must be present in sufficient quantities to ensure effective functioning [13,14,15,16,17]. Nevertheless, ECM development is subject to constant adaptation in response to factors such as chemicals or the environment. The next section will discuss the mechanical signals that result in ECM remodelling [4,18]. Figure 1 shows the primary ECM networks and their constituent elements in different tissues.

As shown by Karamanos et al. [19], proteoglycans have a protein core with negatively charged glycosaminoglycans (GAGs), including heparan sulphate (HS), heparin (Hep), dermatan sulphate (DS), keratan sulphate (KS) and chondroitin sulphate (CS). As outlined, PGs have two main functions: structural and biological. The former is their resistance to compression and hydration of tissues, and the latter their ability to trap growth factors (GFs) in the extracellular matrix [19]. The classification of PGs is based on their location and similarity. There are four groups. When secreted extracellularly, PGs can be divided into two categories: hyaluronan (HA) and lectin-binding PGs (collectively termed hyalectans), and the small leucine-rich PGs (SLRPs). SLRPs control the tissue’s spatial properties. These elements are involved in development and homeostasis. They immobilise GFs within the ECM and regulate collagen fibrillogenesis [20,21,22]. Hyalectan versican can bind to HA and modulate signal transduction mechanisms and cellular processes [23]. Pericellular PGs, perlecan and agrin can interact with various receptors to influence the cardiovascular and musculoskeletal systems [24]. Iozzo et al. [22] show that the combination and arrangement of collagen XVIII and collagen XV, which contain GAG chains, is characteristic of pericellular proteoglycans (PGs) and is observed in all vascular tissues and basement membranes. Proteolytic processes involving the core of perlecan can yield a variety of fragments.

Endorepellin is of particular significance due to its significant antiangiogenic properties [25,26,27]. After being cleaved by proteases and cathepsins, a laminin G-like domain is released, which can bind to a2B1 integrin [28,29]. Endostatin, an anti-angiogenic and antitumoral fragment, is secreted from collagen XVII by several proteases, notably cathepsin L and elastase [30]. Endostatin may be a marker of blood vessel formation. Levels increased after autologous cell therapy in diabetic patients, compared to percutaneous transluminal angioplasty [31]. Researchers have found that certain proteins on cell surfaces play a key role in signalling [32,33,34,35,36]. One of these proteins, GPC-6, appears to be vital for regulating the length of the intestines during embryonic development. By controlling the availability of a protein called Wnt5a, it helps to regulate another protein called Patched1, which in turn affects the two main signalling processes involved in this process [37]. Serglycin (SRGN) was first found to be an intracellular PG, but is now known to be a secreted complex in the matrix [38,39]. As the literature shows, SRGN is involved in the processing and bioavailability of significant molecules, as well as a range of functions, including the maturation of granules and the apoptosis of mast cells, as well as immune modulation [38,39,40].

The hypothesis is that a GAG with distinct functions is HA, which contributes to water accumulation in tissues and their maintenance of shape and form. The subject plays a pivotal role in a number of processes. The function of HA depends on its dimensions, proportion in the sample and capacity to bind with cell receptors and ECM adhesion molecules. Evidence that the HPA axis can regulate signalling supports the hypothesis that its function is context- and tissue-specific. Specific hyaluronan synthases (HASes) catalyse the process of hyaluronan synthesis (HA synthesis) [41,42,43]. Furthermore, HASes are also tissue-specific and can generate HA of varying sizes. The catabolism of HA is a complex process governed by various biological factors, including hyaluronidases (HYALs; predominantly HYAL1, HYAL2, and PH20), reactive oxygen species (ROS), and nitric oxide synthase (NOS). HA engages with several receptors, including cluster of differentiation 44 (CD44), HARE (also referred to as stabilin-2), LYVE-1, the receptor for hyaluronan-mediated motility (RHAMM), also known as CD168, and layilin. It has been demonstrated that HA can interact with these receptors, which can subsequently lead to the binding of other ECM molecules and the activation of function-targeted signal cascades [44,45]. The interplay between HA and CD44 has been demonstrated to induce receptor agglomeration, a process exemplified by the association of Toll-like receptor 4 (TLR4) [46]. As shown by Leng et al. [47], muscle development is governed by the interaction of HA binding to CD44 and RHAMM, which guides myogenic progenitors during migration and growth. Articular cartilage accrues an archetypal ECM, characterised by substantial hydrated aggrecan-HA aggregates, which contribute to its structural integrity [48,49]. HA facilitates the interaction of aggrecan with cell membranes and has been observed to interact concurrently with HAS (CD44) through binding.

Collagen is the main component of ECMs, accounting for over 30% of the total. Collagen types I, II and III make up 80–90% of the total body collagen. Collagens have a distinctive triple-helix structure made up of homo- or hetero-trimeric α-chains. Collagens fulfil two functions: they act as supportive tissue material and ensure elasticity and stability. The collagen family is made up of 28 members. They are divided into subfamilies based on their structures and functions [50,51,52,53,54]. A number of collagens are found in many tissues. Type I and type III collagens often co-distribute. They are types VIII and VI. Collagen IX is present in connective tissues and is often found with collagen II. Some collagens (XI, XXIV, XXVII, XII, XIV, XX, X) are found in connective tissues (tendons, cartilage). Others (XIII, XVII) are found in epithelial tissues. Some collagens are structural components of BM, while collagen type XXVIII is present in the BM of glial cells within the peripheral nervous system. Collagen type XXII is characterised by a highly localised presence in myotendinous junctions [53].

Arteries, lungs and skin are abundant in elastic fibres. This property is important for the circulatory system, where it facilitates uninterrupted blood flow and maintains consistent pressure. Tropoelastin (TE) facilitates the formation of a meshwork of elastin through cross-linking processes, supported by fibrillins and other microfibril protein structures [55,56]. Fibrillins also facilitate the binding of proteins to elastin. Cell signalling is facilitated through interaction with syndecans and integrins. Storage of the transforming GF β (TGF β) family of GFs within the matrix is a critical component of the regulatory network, ensuring optimal functionality [57,58]. Elastin changes during development and childhood, and subsequently experiences a progressive decline in structural integrity during adulthood and ageing [59]. Elastases, a family of proteases, cleave peptides derived from elastin (EDPs), influencing signal transduction. This maintains arterial function, coagulation and prevents skin photoageing [60,61,62].

Lysyl oxidase (LOX) and LOX-like (LOXL) proteins stabilise networks by initiating the initial stage of covalent cross-linking of TE and collagen fibrils [63,64]. They also act as modulators of signal transduction through interactions with various growth factors (GFs), including fibroblast GF 2 (FGF2) and transforming growth factor beta (TGFb), as well as platelet-derived GF b (PDGFRb). In pathological situations, the expression levels of LOX and LOXL can change [65]. Various factors influence the regulation of these processes, including ECM proteins, inhibitors, and PGs. For instance, fibromodulin and syndecan-4 have been demonstrated to facilitate the crosstalk between LOX proteins and collagens [66,67]. Thrombospondin-2 has a regulatory influence on the skin’s elastic qualities. This is evidenced by observations that its gene expression can attenuate collagen fibrilogenesis and reduce LOX levels [68,69,70]. Fibronectin is another macromolecule that forms supramolecular assemblies. It regulates mechanical properties, such as tension, by changing its fibres’ conformation. Fibronectin also interacts with integrins, which regulate cellular adhesion, as well as with GFs, cytokines and ECM molecules [71,72,73].

The laminin family comprises over 16 members, each with three chains. The distribution of laminin molecules exhibits specificity to tissue type and cellular origin. Laminin-111 is predominant in embryos, while laminins 521 and 511 are predominantly present in mature tissues. Laminins 211 and 221 demonstrate a more specific distribution. Laminins 211 and 221 have a more specific distribution. Laminins 411 and 421 have been detected in the basement membrane (BM) of endothelial cells, while laminin-332 has been found in the BM of the epithelium. In contrast, laminins 411 and 421 have been found in the BM of skeletal and cardiac muscles [74,75,76,77].

The tenascin (TN) family is categorised as a matricellular protein. It has four distinct members: TN-C, TN-R, TN-W and TN-X. TNs have three domains: EGF-like, fibronectin-type III and fibrinogen-like. These domains interact with many other ECM proteins, including collagens, fibronectin, fibrillins, PGs, GFs, chemokines and soluble factors. TNs regulate cell adhesion via integrins, playing pivotal roles in embryonic development, pathogenesis and tissue homeostasis [76]. TN-C is implicated in tissue formation and regeneration, and its presence in adult tissues is restricted to specific stem cell populations, lymphoid organs and tendons [78,79]. TNs regulate cell adhesion via integrins, playing pivotal roles in embryonic development, pathogenesis and tissue homeostasis [76]. TN-C is implicated in tissue formation and regeneration, and its presence in adult tissues is restricted to specific stem cell populations, lymphoid organs and tendons [78,79]. This process orchestrates tissue repair. However, persistent high TN-C expression can promote pathologies, including inflammation, fibrosis, myocarditis and cancer. TN-X plays an integral role in organogenesis, being present in late embryos throughout development [80,81,82]. TN-W is implicated in osteogenesis and is found in specific stem cell niches and dense connective tissues. TNR is expressed in the CNS and linked to neurogenesis [83,84,85,86,87].

This review describes the structural organisation and functions of basement membrane (BM) and loose connective tissue in the section of the ECM as tissue-distinctive functional meshwork. It covers the main types of secreted PGs found on the cell surface, inside the cell, and in BM/pericellular space. The text looks at the GAG parts of these PGs, where they are found in the body, and their role in the matrix. GAGs, which work with the ECM, are the branches of the PG tree and are very different in structure and have different biological functions.

**Figure 2:**
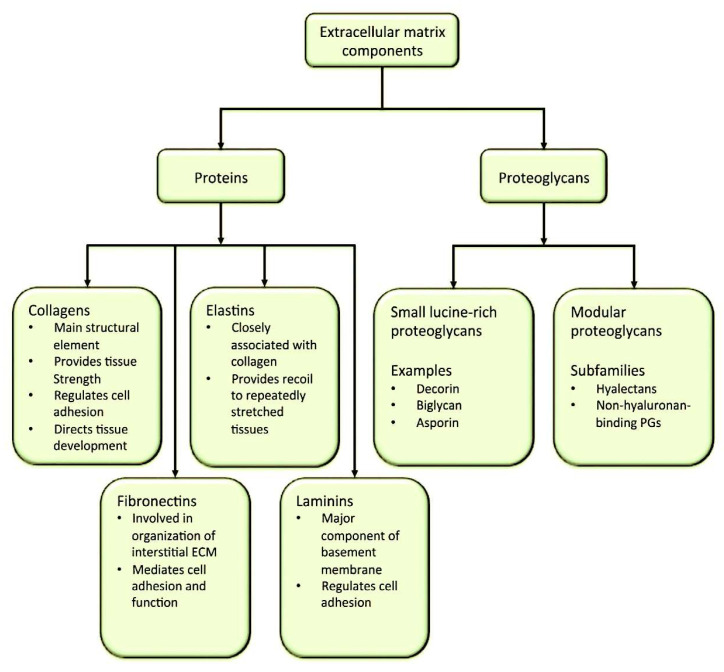
The ECM comprises two primary classes of biomacromolecules, namely proteins and proteoglycans. The ECM is composed of four primary protein types: collagen, fibronectin, elastin, and laminins. Notes: Notes: Proteoglycans represent a broad class of molecules that can be subdivided into three main subclasses: small lucine-rich proteoglycans, modular proteoglycans, and cell surface proteoglycans. It has been established that both classes of macromolecules play a crucial role in the structural and signalling processes.

The BAV is explored in detail, and the thesis focuses squarely on hyaluronan, addressing its synthesis, degradation and functions. Hyaluronan receptors, which play a role in intracellular signalling, are presented separately. The following sections will address the critical roles of specific ECM macromolecules in the structure and function of the ECM. These include elastic fibres, which are essential for tissue elasticity, and laminins, which act as adhesion proteins. Integrins will be discussed as adhesion and signalling mediators between the ECM and cells, along with their ligands and activation mechanisms. Consider the roles of these factors in the context of pathophysiology. The final section of this compendium examines the functions of the critical enzymes involved in tissue remodelling and human disease as well as the BAV. This is the most common heart problem in people born with it, affecting 1–2% of the general population. In BAV the fibrillin-1 gene may be structurally intact, but its regulatory elements may be compromised. Transcription elements are significant in many congenital cardiac pathologies. Another important factor is the gene that governs nitric oxide synthesis, which is important for the cardiovascular system [1,4]. BAV-associated complications require intervention. The literature repeatedly notes that healthcare practitioners often have difficulty providing evidence-based counsel on BAV disease. This is due to the current state of knowledge regarding the genetics, molecular regulation, pathogenesis and pathophysiology of the condition, which remains limited and less understood compared to other areas of medicine [88,89,90,91,92,93] (Figure 2).

### 1.2. The Structure and Function of Basement Membranes and Connective Tissue

**Figure 3:**
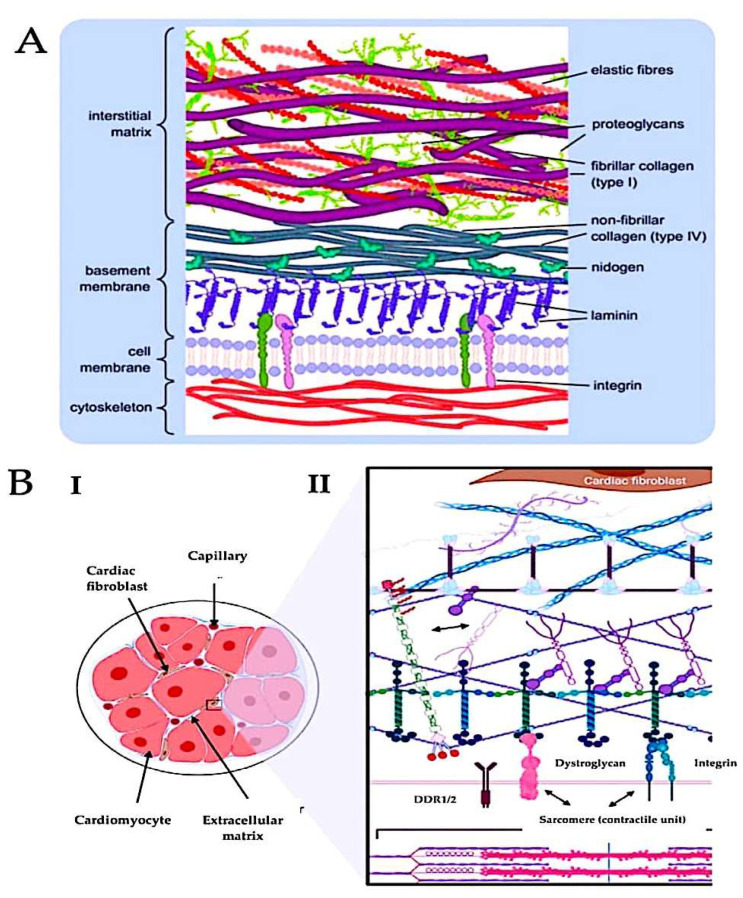
(**A**). The simplified extracellular matrix structure of a vascular system is defined as a three-dimensional macromolecular network composed of various proteins and polysaccharides. The pericellular matrix constitutes a layer in close proximity to the cells. Integrins bind to polymerised laminin, which, in turn, is connected via nidogen to the type IV collagen. Interstitial matrixes form a porous network comprising fibrillar collagens, elastic fibres, and proteoglycans. (**B**). (**I**) The basement membranes within the heart are of particular interest. The heart’s ECM comprises the BM and the interstitial matrix, which contains fibrillar collagens, such as collagen I and collagen III. The basement membrane (arrow blue) envelops the cardiomyocytes and microvasculature. Interspersed within the ECM are cardiac fibroblasts (arrow brown), which are responsible for the secretion of numerous matrix components. (**II**) A magnified view of the BM as an interface between the interstitial matrix and the sarcolemma of the cardiomyocyte is presented herewith. It has been established that a variety of BM components interact with the cardiomyocyte sarcolemma via integrin receptors, dystroglycan receptors and DDRs. Within the BM, laminin and collagen IV networks are organised in a complex manner, with perlecan and nidogen acting as ‘bridging molecules’ between these networks. Collagen XV and XVIII exhibit their characteristic polarisation, thereby establishing a linkage between the BM and the interstitial matrix. Collagen VI microfibrils interact with collagen IV, thereby establishing a physical linkage between the BM and the fibrillar components of the interstitial matrix. For the sake of comprehensibility, the lateral accumulation of collagen type IV is not displayed, and only a few illustrations of BM constituents and their interrelationships are provided. Refs. [1,2,3,17,20].

The classification of ECMs is dependent on their topographical position, with pericellular matrices enhancing cellular processes. Attachment and an interstitial matrix providing tissue integrity are required. The former is a closely structured network that comes into contact with cells, forming cross-junctions via integrins, discoidin domain receptors (DDRs), and proteoglycans (PGs), such as syndecans [2]. A paradigmatic exemplar of pericellular ECM is the BM, which is abundant in laminin isoforms and collagen IV (Figure 3).

Self-assembled networks are created by both of them and are connected through ninogens and the HSPGs, perlecan and agrin. An interactive and supportive microenvironment for the local cells is created by these components, with laminins acting as tethers with the local cytoskeleton [13,94,95]. Laminins have two functions: they provide a framework and they send signals. The architecture of BM is defined by the interaction of their N-terminal domain with several BM biomacromolecules. The C-terminal domain of the protein binds to cell membrane receptor sites, including a3b1, a6b1, a6b7, and a7b1 integrins. This establishes a connection point for various biochemical signals and the cells, allowing processes such as adhesion, migration, survival, differentiation, and apoptosis to occur [96,97]. Cell signalling is affected by integrins through a bridge they create linking the external ECM and the internal cytoskeleton [98,99]. It was revealed by a recent study that crucial functions such as adhesion, proliferation, and migration of kidney epithelial collecting duct cells are carried out by the cooperation of a3 and a6 integrin subunits [100,101]. A greater reduction in these properties is caused by the concurrent deletion of these subunits, and a bigger one is caused by the concurrent deletion of these subunits.

The signalling cascades are impaired and the cellular reaction to FGF-10 and glial cell line-derived neutrophilic factor is minimised, unlike the milder alterations observed upon the deletion of a single subunit [101]. BM contains tissue-specific functions and the following matricellular proteins: secreted protein acidic and rich in cysteine, cartilage oligomeric matrix protein (COMP), thrombospondins (THBSs), tenascin (TN), pigment epithelium-derived factor (PEGF) and osteopontin (OPN).

Tissue homeostasis is also played by these proteins. The BM is underpinned by the interstitial connective tissue that surrounds, separates and supports other tissues. The composition of its ECM can vary, with the aim of creating specific environments for distinct tissue functions. Loose, dense and specialised connective tissues can be distinguished. Collagens are resistant to tensional loads. This is mainly type I, but also type III. Elastin may further support tissue stretching. PGs, on the other hand, are present in these ECMs in large quantities and act in opposition to compression forces. Specialised matrix components, such as PGs and proteins, enhance the tissue’s mechanochemical properties. Specialised ECM is found in bone and cartilage. Cartilage ECM mainly comprises a viscous gel that is rich in avascular PG aggregates, while bones constitute stiff, mineralised, and vascularised ECM.

### 1.3. The Role of Macromolecules in the Composition of ECM

#### 1.3.1. The Function of Proteoglycans: Key Components in the Organisation of the ECM and Cell Processes

Proteoglycans have two main functions: regulating matrix structural organisation and mechanical properties and integrating major signalling cascades that govern cell behaviour [19,20,21,22,102,103,104,105,106,107]. PGs exhibit various characteristics in cell behaviour and signalling due to their function as coreceptors for GFs and their ability to facilitate chemokine signalling through G protein-coupled receptors (GPRs) [108,109,110]. Numerous studies demonstrate a clear correlation between PG expression and the properties of integral cells [111,112,113]. Altered PG expression and post-translational modifications in cancer cells and tumour stroma significantly impact cancer progression and response to therapeutic interventions [114,115,116].

There is a single intracellular PG, SRGN, which is a constituent of the secretory granule. This PG is found within HS chains and contributes to mast cell secretory granule formation. It also plays a regulatory role in the storage of GFs and cytokines [38,39,40]. The phenomenon is expressed in inflammatory, endothelial, and smooth muscle cells, Ref. [114] as well as in tumour cells [40]. SRGN is necessary for the storage of proteases in mast cells and for the storage of granzyme B in T-lymphocytes. It has also been shown to play a role in cell granule-mediated apoptosis [114]. As is well documented, this PG has been shown to stimulate the release of inflammatory mediators, promote tumour growth and facilitate progression [115,116,117].

PGs, hyalectans and small LRPs (SLRPs) have been found in interstitial ECMs, where they interact with other ECM constituents, ensuring stability. HLA interacts with PGs through HA binding within its C-terminal domain. Hyalectans comprise four components: versican, aggrecan, brevican and neurocan. Versican is a multifaceted molecule with isoforms V0, V1, V2 and V3. It is widely distributed across tissues, suggesting a critical role in physiological processes. It is primarily expressed in the brain, lungs, female tissues, heart, smooth muscle and adipose tissue, with secretion into the blood being a major feature [118]. Scientific investigation has determined that the protein exhibits a dynamic interplay with calcium and HA, contributing to critical processes such as cell adhesion, proliferation, migration and angiogenesis. This protein is key to tissue morphogenesis and homeostasis [38,119,120]. Aggrecan is a component of the ECM in cartilage and exhibits compressive resistance. It is found in many tissues, including the brain, lung, seminal vesicles and cervix, as well as soft connective tissue [118]. This PG is heavily glycosylated by CS and KS, with many potential GAG attachment sites. Brevican and neurocan are CS PGs that are specifically overexpressed in the CNS, particularly in the brain cortex, and interact with HA [118].

The largest class of PGs, which is characterised by the presence of small leucine-rich proteoglycans, is made up of the most abundant matrix components (18 members). These components possess distinct gene products that are responsible for carrying out a variety of significant biological processes. The family under discussion interacts with growth factors (GFs), cytokines, receptor tyrosine kinases (RTKs), and toll-like receptors (TLRs). Through this interaction, vital processes such as embryonic development, homeostasis, migration, proliferation, angiogenesis, innate immunity, apoptosis, and more are regulated. Autophagy [103] is also involved in these interactions. SLRPs have been identified in all interstitial matrices, where they can act as structural constituents and signalling molecules, especially during ECM remodelling in conditions like cancer, diabetes, inflammation, and atherosclerosis. Research shows decorin, lumican, fibromodulin and biglycan are involved in cell signalling, the mesenchymal-to-epithelial transition (MET), and processes like inflammation, autophagy, collagen fibrillogenesis, and matrix architecture [121,122,123,124]. The third category of extracellular PGs comprises the SPOCK/testican family, which includes three calcium-binding HSPGs found in the interstitial ECMs. SPOCK1 is exclusively located in the cerebral cortex, SPOCK2 in the brain, and SPOCK3 in the lung and endocrine tissues. SPOCK3 transcript levels have been measured. SPOCKs are found in the parathyroid gland [118]. SPOCKs have been linked to neuronal mechanisms within the Central Nervous System (CNS) [125,126].

The pericellular/BM PGs are a group of proteins that include perlecan and agrin, as well as collagens XVIII and XV. These proteins are linked to cell surfaces via integrins or other signalling molecules. They can also form part of the largest BMs [127]. These proteins have a large protein core and many structural motifs. The C-terminal domains of perlecan and collagen XVIII have been shown to have both anti-angiogenic and autophagy effects after being processed [30,128]. These pericellular/BM PGs interact with RTKs and other cell surface receptors, as well as other matrix molecules. These interactions control cell signalling, migration, angiogenesis, autophagy, matrix assembly and vascularization [22]. Perlecan can transport up to three HS/CS chains and is found in smooth muscle tissue, the endometrium, the urinary bladder and adipose tissue. Agrin, found in the kidneys, gallbladder and skin, has three HS chains bound to it. Collagens XVIII and XV are found in the liver, brain, female tissues, heart muscle and adipose tissue. However, the highest levels of expression are in the bone marrow (BM). This suggests that these tissues may be important in BM adhesion to the surrounding collagenous connective tissue matrix [118]. Cell surface PGs interact with cell receptors (e.g., epidermal GF receptor (EGFR), HER2, TGFbRI, TGFbRII, insulin-like growth factor receptor I) and integrins (amb3, amb5, a3b1, a6b4, a5b1). These interactions facilitate cell signalling [129,130,131,132,133].

Glypicans have undergone a modification involving HS chains in the juxtamembrane region, and are secured to the plasma. The membrane is linked via a C-terminal glycosylphosphatidylinositol (GPI) linkage, the cleavability of which is facilitated by the lipase Notum. Six GPCs (GPC1-6) are present in mammals, predominantly exhibited in epithelial and mesenchymal cells, and regulate cell proliferation and developmental processes. GPCs have also been identified as potential cell membrane proteins that can interact with GFs. The following GFs have been implicated: Wnt/b-catenin, Hh, fibroblast GF (FGF), insulin-like GF (IGF), and VEGF. TGFb and matrix-modifying enzymes, encompassing proteases and lyases, are facilitated through their HS chains in a multitude of cancer cells. These processes are thus pivotal in cell signalling and the regulation of tumour growth, angiogenesis, and metastatic spread [134,135,136,137,138,139,140,141,142,143,144,145,146,147,148] sustained by cell migration [149,150,151,152]. Migration instigates the motility of endothelial cells during microvascular morphogenesis [149].

GPC1 is found in the CNS, skin, and skeletal system, but studies have found higher levels in breast, pancreatic, prostate, pancreatic, and oesophageal cancer cells [136,137,138,139,140]. GPC2 is important for neuronal cell adhesion and neurite outgrowth, and is linked to poor survival in neuroblastoma cases [141,142].

Betaglycan/TGFb type III receptor is a soluble transmembrane proteoglycan (TM PG) with an HS/CS chain that is a constituent of the TGFb superfamily of coreceptors [153,154]. Its extracellular domain contains GAG attachment sites and protease-sensitive sequences located near the plasma membrane. It is a cell surface PG widely expressed in testicular, breast and ovary cells [118]. Phosphacan (PLCB) is a type of protein tyrosine phosphatase b (PTP-β) expressed exclusively in the cerebral cortex of the brain [134]. It consists of up to five CS/DS chains. It has been posited that phosphacan exerts a regulatory role in specific developmental processes within the CNS [155,156,157].

#### 1.3.2. The Role of Glycosaminoglycans to Modulate the ECM

PGs are proteins that serve as cores, into which one to over a hundred GAG chains are covalently anchored. The structural heterogeneity of disaccharide units and sulfonylation levels is a hallmark of six defined GAGs: CS, DS, Hep, HS, KS and HA [158,159]. GAGs are linear heteropolysaccharides with a repeating disaccharide structure of hexuronic acid (D-glucuronic or L-iduronic acid) or galactose only in KS, and N-acetylated hexosamines (N-acetyl-D-glucosamine). They are unbranched and negatively charged. Alternatively, the substance is referred to as N-acetyl-D-galactosamine. With the exception of HA, GAG biosynthetic processes take place in the endoplasmic reticulum and Golgi apparatus via distinct biosynthetic pathways that are overseen by various enzymes [19]. Hyaluronic acid (HA) is the most basic GAG in nature and the only GAG. It is synthesised on the cell membrane and quickly released into the extracellular environment with no covalent attachment to proteins. The structural and functional diversity of pro-teoglycans is due to the different saccharide units, dimensions and sulfonylation sites in the disaccharide components of the repeating structural element, as well as the many attachment sites for GAGs to core proteins [159,160].

Hep and HS exhibit elevated sequence heterogeneity and divergent sulfation patterns. Consequently, the preponderance of GAG-binding proteins interact with these GAGs [161]. HS exhibits a reduced tendency towards sulfation and C-5 epimerization of D-glucuronic to L-iduronic acid. It plays a pivotal role in maintaining homeostasis, facilitating embryonic growth and contributing to pathological processes through its engagement with GFs, cell surface molecules and matrix enzymes. This interaction modulates critical signalling pathways [159,162,163]. The regulatory processes governing the impacts of HS on GF signalling are modulated by heparanases (HPSE), sulfotransferases and sulfatases [164,165,166,167]. In addition to its established anticoagulant properties, there is mounting evidence from multiple sources that Hep and nano-Hep derivatives have potent anticancer activity across various tumour types [168,169]. Hep and nano-Hep derivatives have been shown to impact breast cancer cell proliferation and metastasis in models of both in vitro and in vivo. These derivatives have also been shown to regulate the profile of major ECM macromolecules, thereby offering evidence for therapeutic targeting [170,171].

The SARS virus, which causes the common cold, can bind to and enter cells using a process involving the angiotensin-converting enzyme 2 (ACE2) receptor. This process is called viral attachment and infection and depends on a specific human protein (HS). The receptor-binding domain has been shown to facilitate this process. In addition, as demonstrated by Clausen et al. [172], stopping the virus from attaching to and infecting cells using Hep and nonanticoagulant derivatives offers a new way to treat patients with severe acute respiratory syndrome caused by the novel coronavirus (SARS-CoV-2). The constituent sugars of KS are classified as disaccharides, which can be non-sulphated, mono-sulphated or di-sulphated, like HS. KS is expressed and the protein plays a role in wound healing, embryogenesis, collagen fibre assembly, and corneal organisation in cartilage and epithelial and neural tissues [173,174]. It has also been detected in neurosecretory vesicle membranes, indicating its potential involvement in vesicle biogenesis and neuronal function [175].

## 2. The Collagen in the Organisation of the ECM

Collagens are a family of ECM proteins that share common characteristics. The triple helix is a structural motif. There are twenty-eight collagen types, numbered by Roman numerals (I–XXVIII) [176,177] and (IX, X, XII, XIV, XVI, and XIX) [178,179,180,181,182,183,184,185,186,187,188,189,190,191,192,193,194,195,196,197,198,199,200,201,202,203,204,205,206,207,208,209,210,211,212,213,214,215,216,217,218,219,220,221,222,223,224,225,226,227,228,229,230,231,232,233,234,235,236,237,238,239,240,241,242,243,244,245,246,247,248,249,250,251,252,253,254,255,256,257,258]. Collagens provide the structural organisation and mechanical properties of the extracellular matrix and tissues. Collagens also modulate a plethora of biological processes by engaging with cell surface receptors. This regulatory function is exercised by collagens as full-length proteins or through their bioactive fragments, known as matricryptins or matrikines, which are released via limited proteolysis. Alterations in collagen synthesis, accumulation, cross-linking, and/or breakdown have been observed in numerous pathological conditions involving compromised stromal collagen organisation, including neoplasms [183,184,185,186,187] and fibrotic diseases [188,189,190,191,192,193,194]. Furthermore, mutations within the gene sequences that encode collagens have been demonstrated to be associated with a multitude of genetically mediated pathologies [195,196].

Recent studies have highlighted the importance of the local sequence in the mechanics and structural stability of the triple helix. This raises the question of sequence-specific mechanical stability and function [52]. Other proteins, such as soluble defensive collagens and membrane proteins, also have a triple helix and are therefore part of the collagen superfamily. A significant proportion of collagen types have non-collagenous domains, emphasising the complexity and diversity of this protein family. Examples of such domains include fibrinogen III, von Willebrand factor A, thrombospondin, and Kunitz inhibitor domains [197]. Most collagen types form supramolecular assemblies with other elements of the extracellular matrix. Collagens self-assemble into fibrils and networks, which anchor fibrils and generate filaments [198,199]. Collagen VI forms networks with collagen IV, VIII and X, but certain collagens do not form supramolecular structures. These entities can self-assemble and form associations with extant ones, such as the FACITs.

### 2.1. Fibrillar Collagens

The most common types of fibrillar collagen are I, II, III, V and XI, with few studies focusing on XXIV and XXVII. Collagen I is found in many tissues, collagen III in cartilage, collagen V in tendon, and collagen XXIV in bone [204,205,206]. It promotes bone formation via the TGFb/Smads signalling pathway [207,208]. Overexpression of COL24A1 in liver cancer predicts poor prognosis [209]. Collagen XXVII plays a pivotal role in the organisation of the pericellular matrix within the growth plate [210,211]. Genetic variations in COL27A1 have been identified as a causative agent in Steel syndrome [212].

As Kuivaniemi et al. have shown, different collagens form different 3D structures, depending on the tissue type and the particular collagen type [214]. The process of collagen fibrillogenesis is meticulously regulated at the cellular level, depending on the tissue type and the developmental stage [198]. This regulatory mechanism is orchestrated by the FACITs, the ECM protein fibronectin, and SLRPs [199]. Suppression of the circadian clock has been demonstrated to induce an aberrant collagen response, with fibrils and collagen accumulation [199]. A circadian clock mechanism of protein homeostasis has been identified, with nocturnal procollagen synthesis and daytime collagen fibril assembly [215]. Collagen fibrils are stabilised by covalent cross-links, which give the ECM and tissues their mechanical properties (i.e., resistance to traction). Covalent cross-linking of fibrillar collagens is initiated by lipoxygenases (LOX) [65], which catalyse the oxidative deamination of specific lysine and hydroxylysine residues into aldehydes [216]. These aldehydes react with other lysine and hydroxylysine residues to form cross-links. Glycation leads to the formation of glycation end products, which form cross-links in collagens [217].

Hydroxylation of collagens occurs post-translationally, influenced by proline and lysine. Gjaltema et al. and Ishikawa et al. highlight the importance of hydroxylases in this field [218,219]. Research shows that collagen IV is more glycosylated than fibrillar collagens [220,221]. Mass spectrometry can be used to identify post-translational collagen modifications [222]. Heat shock protein Hsp47 prevents collagen misfolding in mammalian cells [220]. It regulates collagen I proteostasis [221]. This investigation focuses on mutations in collagen genes. I, III and V are linked to Ehlers-Danlos syndrome, a group of hereditary disorders. These features include joint hypermobility, skin that is soft and easily bruised [223]. Genetic mutations can contribute to the development of skeletal disorders. Research has indicated a correlation between COL2A1 and skeletal disorders [224].

### 2.2. Network-Forming Collagens

Collagens IV, VIII and X are structural proteins that play a crucial role in bodily functions. Collagen IV is found in basement membranes. These proteins are linked via networks formed at their N and C termini [225,226]. Crosslinking of these networks is stabilised by LOX-2 and peroxidasin. The sulfilimine cross-link formed by peroxidasin contributes to kidney tubular BM stiffness [227] and collagen IV scaffold formation [228]. Mutations in the genes COL4A3, COL4A4 and COL4A5 cause Alport syndrome, a hereditary kidney disease [229]. Genetic variation in the COL4A1 gene has been shown to cause hereditary angiopathy, nephropathy, aneurysms and muscle cramps syndrome [230].

A specific type of Bruch’s membrane contains collagen VIII, arranged into hexagonal lattices. Endothelial cells and vascular smooth muscle cells synthesise it, and COL8A2 regulates corneal endothelial cells [231]. Collagen VIII is increased in atherosclerosis [232] and reduced in heart failure [233]. Collagen X is restricted to the hypertrophic zone of the growth plate, ref. [234] regulating endochondral ossification and haematopoiesis [235]. COL10A1 expression is increased in tumours and associated with tumour vasculature [236]. Mutations in the COL10A1 gene are associated with Schmid-type metaphyseal kondrodysplasia, a rare inherited disorder characterised by short stature, short limbs and bowed legs [237].

### 2.3. Stick-like Collagen-Forming Fibrils

Collagen VII creates anchoring fibres at the dermal–epidermal junction. When collagen VII is lost, this causes inflammation, which leads to scarring [238]. Collagen VII accelerates wound healing by promoting closure and facilitating cell migration. It also regulates the healing process of wounds. The body synthesises cytokines within the tissue where new blood vessels are formed [239]. Collagen VII is also found in podocytes of normal human kidneys and in endothelial cells of the glomerular filtration barrier [240]. Changes to a gene called COL7A1 can cause epidermolysis bullosa, where the body makes antibodies that attack collagen VII, which can lead to the skin splitting [242].

## 3. Elastic Fibres and Elastin Function to Preserve Tissue Elasticity at the ECM Level

“Elastic fibres” are responsible for the necessary elasticity and extensibility of the body. These elements facilitate the functions of numerous organs, including arteries, skin, tendons and lungs. The material is capable of undergoing reversible deformation and repeated mechanical stress. Research has revealed that the composition of elastic fibres consists of two morphologically distinct entities: a longitudinal mantle, in conjunction with the alignment of fibrillin-based microfibrils and a dense core. The material is predominantly composed of cross-linked elastin, accounting for over 90% of its composition. Li et al. [259] state that microfibrils are filaments measuring 10–12 nanometres wide and resembling uniform, perfectly spherical beads arranged in parallel. Elastin deposition on a microfibrillar scaffold facilitates long-range elasticity in tissues, a process that is particularly pronounced in the presence of elastin. Microfibrils are primarily composed of fibrillins [260], though other associated proteins include microfibril-associated glycoproteins (MAGPs) [261], elastin microfibril interfaces, disintegrin [262] and metalloproteinase with thrombospondin motifs (ADAMTS) and ADAMTS-like proteins [263,264], and latent transforming growth factor beta (LTB) binding proteins (LTBPs, such as LTBP-4) [265].

The other principal constituent, elastin, is an insoluble biopolymer composed of individual units of its soluble precursor form, TE. TE’s primary structure is characterised by the alternating presence of hydrophobic and hydrophilic domains, encoded by distinct exons. The domain structure of TE maps the exon organisation of the gene. Hydrophilic domains containing lysyl-alanine (KA) or lysyl-proline (KP) motifs have been demonstrated [266,267]. These motifs are involved in covalent cross-linking, which leads to elastin maturation. Hydrophobic domains are responsible for elasticity and cell interactions [268]. The primary transcript of elastin shows high levels of non-canonical splicing, producing many isoforms without affecting the reading frame. Studies [269,270] have shown some of these isoforms are present in human tissues.

Mature elastin is stable over an organism’s lifespan when there are no underlying pathologies. Its half-life is greater than 70 years [271,272]. This is due to its high resistance to proteolysis from its cross-linking structure and dense packing. Elastin is hydrophobic and insoluble, but its ability to hydrate is vital for its elasticity [273,274]. Gene expression of fibrillins marks the start of elastogenesis, with these subsequently assembling into microfibrils. These microfibrils then serve as a structural framework for the deposition of TE [275]. The fibrillin network has been observed to undergo cross-linking, a process that has been shown to stabilise the three-dimensional bundle structure. The literature on the subject shows that cross-links can be of two kinds: intermolecular disulfide bonds [276,277] and e(c-glutamyl) lysine cross-links, the latter being catalysed by members of the transglutaminase family [278,279,280].

**Figure 4:**
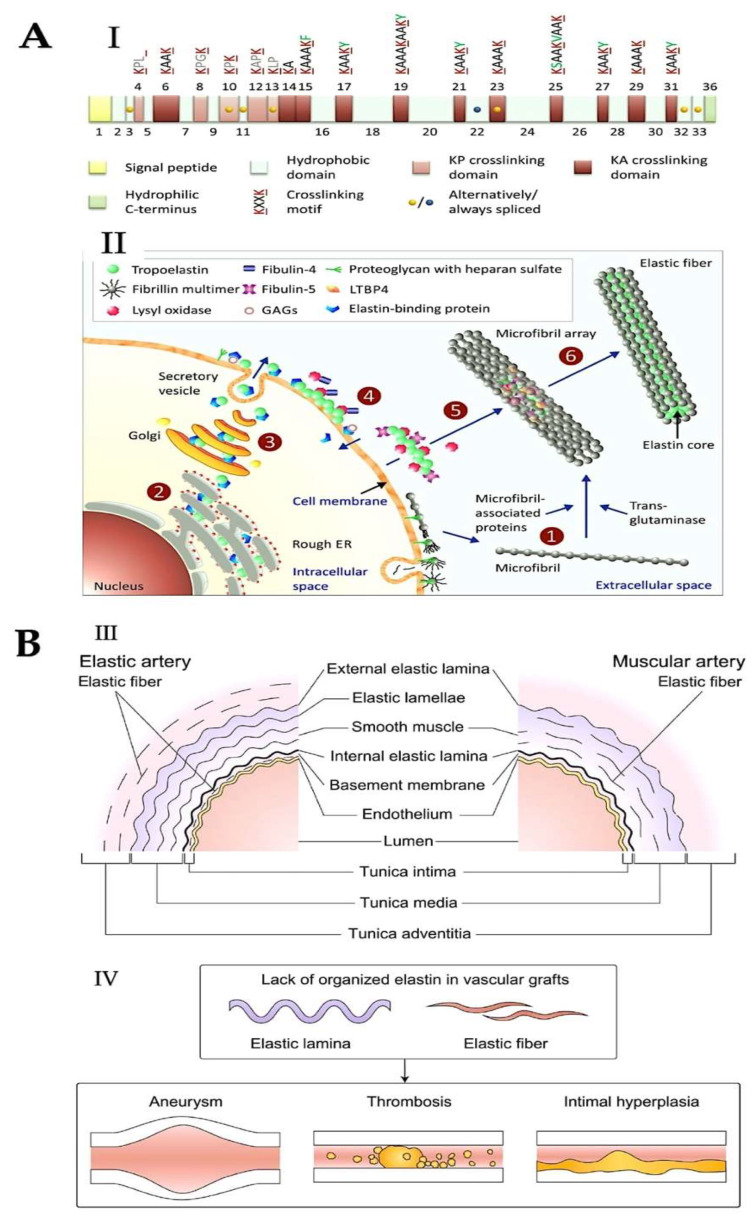
(**A**); (**I**), Schematic representation of the exon and domain structure of human TE. The numerical sequence is determined by exon assignment and serves to denote crosslinking domains (indicated by the numbers at the top; light and dark red squares) and hydrophobic domains (indicated by the numbers at the bottom; grey squares). Domains that have been subject to encoding by exons, and that are thus open to alternative splicing, are indicated by filled circles. The arrangement of sequence patterns within the eleven Lys-Ala and five Lys-Pro crosslinking domains is illustrated above the scheme. The width of the squares is proportionate to the respective domain sequence length. (**II**), The assembly of elastic fibres. (1) fibrillin and microfibril-associated proteins are secreted into the extracellular space, where they multimerise. The formation of the microfibrillar array is the subject of this study. (2) The process of TE synthesis occurs within the rough endoplasmic reticulum, where it becomes bound to the chaperone EBP. (3) It has been established that the EBP-TE complex is transported through the Golgi apparatus and subsequently secreted to the cell membrane. As a consequence of the interaction with GAGs, EBP dissociates. (4) Concurrently, TE is released from the chaperone and forms globules at the cell surface. Fibulin-4 plays a pivotal role in the alignment of the chain, a process that is critical for the mediation of the interplay with LOXs. The oxidation of Lys residues is followed by various condensation reactions, leading to the formation of covalent intra- and intermolecular cross-links. (5) Once a critical size has been attained by the cluster of TE molecules, it is then moved from the plasma membrane through the extracellular space. It is hypothesised that fibulin-5 directs the premature elastin to fibrillin microfibrils. (6) The elastin aggregates then fuse into larger assemblies with support by LTBP-4 and are subsequently further cross-linked. As the body undergoes various changes throughout the lifespan, elastin is subject to non-enzymatic processes and proteolytic cleavage. The latter process has been shown to result in the release of bioactive peptides, known as elastokines. (**B**); (**III**) The structural composition of the elastic and muscular arteries. It is an observable fact that both types of arteries are composed of tunica intima, tunica media, and tunica adventitia. In the case of elastic arteries, the tunica intima consists of the endothelium and a basement membrane that contains elastic fibres. The tunica media is composed of numerous continuous elastic lamellae, which are separated by thin layers of smooth muscle cells. The tunica media is delineated by the internal and external elastic lamina. The tunica adventitia comprises fibroblasts and elastic fibres. In the case of muscular arteries, the tunica media is characterised by the homogenous distribution of elastic fibres, which may be either scattered or discontinuous elastic lamellae. (**IV**) The absence of structured elastin within these elastic components is a pivotal element that contributes to the development of aneurysm, thrombosis, and intimal hyperplasia in vascular grafts. Abbreviations; EBP; Elastin Binding Protein; ER, Endoplasmic Reticulum; LTBP, Latent TGFb Binding Protein; PTMs, PostTranslational Modifications.

The TE monomer process is observed in elastogenic cells. TE involves the rapid self-association of distinct entities, driven by a heat-driven and entropic mechanism. The resultant state is coacervation, leading to the formation of distinct globular aggregates on the cell membrane [281]. This phenomenon has been investigated further. Proteins may interact with this domain, promoting the accurate arrangement of TE monomers [282,283,284]. TE binding and cross-linking are promoted by fibulin-4 and fibulin-5, facilitating the association between TE and the extracellular Cu^2+^-dependent amine oxidase, LOX [285]. Enzymes LOX and LOXL facilitate the oxidative deamination of the e-amino group of Lys residues, forming α-amino groups. Cross-links are formed through the reaction of allysine, formed from a-aminoadipic acid-d-semialdehyde [266,267,268]. This results in desmosine and its isomer, isodesmosine [266]. Elastin’s prolonged half-life causes molecular ageing, which instigates reactions like oxidation, aspartic acid racemization, glycation and carbamylation [286,287,288]. This molecular ageing of elastin coincides with the ageing process experienced by the organism as a whole. However, it can be exacerbated by certain chronic diseases, including diabetes, kidney disease and atherosclerosis [289,290,291,292]. The repercussions of elastin’s molecular senescence are manifold, affecting the structural and mechanical characteristics of this protein and cellular components [293,294] (Figure 4).

Studies show that elastin is subject to mechanical stress [295,296]. When it breaks down, the functionality of the elastic fibres is reduced, causing a shift in tensile stress from the elastin to other extracellular constituents, such as collagen fibres. This affects tissue mechanics [297]. Furthermore, the rupture of these fibres is exacerbated by the action of members of several classes of extracellular proteases, known as elastases. These enzymes can be categorised into three distinct classes according to their substrate specificity. The first class comprises serine proteinases, which include cathepsin G, proteinase 3, and neutrophil elastase [298,299,300]. The second class includes matrix metalloproteinases (MMPs). The literature says to include MMPs 2, 7, 9, 12, and 14 [301,302], and cysteine proteinases cathepsins K, L, S, and V [303,304,305]. Elastin degradation leads to the secretion of bioactive peptides called elastokines, which are a type of matrikine protein [306,307].

The bioactive EDPs released thus far have been shown to induce various biological effects [306,308,309]. These effects include cell adhesion [310,311], chemotaxis [312], migration [313], proliferation [314], proteinase activation [315], angiogenesis [316], and apoptosis [317]. These elements are implicated in numerous pathophysiological mechanisms, including cancer progression [312,313], emphysema, and the progression of vascular diseases such as aneurysm formation [1,2,3,4]. The EBP subunit [317] has two functional interaction domains: the elastin site (direct role in intracellular signal transmission) and the galactolectin site (releases EDPs and ERC dissociation when occupied by galactosugars) [318]. EDP binding to EBP activates Neu-1, which converts GM3 ganglioside to lactosylceramide (LacCer), an essential second messenger in ERC signalling [319,320]. The human elastin receptor is a 67 kDa protein that binds to both elastin and laminin fragments [316]. It has been established that a ternary complex, termed the elastin receptor complex (ERC), is formed when 55 kDa cathepsin A/protective protein and 61 kDa membrane-bound neuraminidase-1 (Neu-1) interact [316].

EDPs can activate multiple tyrosine kinases in fibroblasts, including MEK1/2 and ERK1/2, through PKA and PI3K mechanisms [321]. In endothelial cells, MAPK signalling is initiated through the PI3K/Akt/eNOS/NO/PKG pathway [322]. In smooth muscle cells, EDP binding to ERC activates Gi proteins, opens L-type calcium channels, and causes cell proliferation [323]. Neu-1 performs a pivotal function in the context of the aforementioned signalling cascades. A substantial body of research has been undertaken to further illuminate its role in ERC signalling in various pathophysiological scenarios [319,324,325,326,327]. Research has demonstrated that a distinctive dimerization process is required for its catalytic activity, in addition to its role in the production of the second messenger LacCer. Two possible dimerization motifs in the human Neu-1 protein have been identified. Point mutations in the 148–168 and 316–333 TM domain have been shown to inhibit Neu-1 dimerization and sialidase activity [328]. Neu-1 has also been shown to regulate the activation of various membrane receptor proteins through desialylation [307,316,329,330,331]. The findings suggest that Neu-1 plays a pivotal role in the membrane signalosome. These signalling pathways are accountable for the impact of elastokines on pathological processes. Research is directed towards identifying mechanisms to impede implicated processes.

## 4. Laminins Fulfil the Role of Adhesion Proteins Within the ECM Network

**Figure 5:**
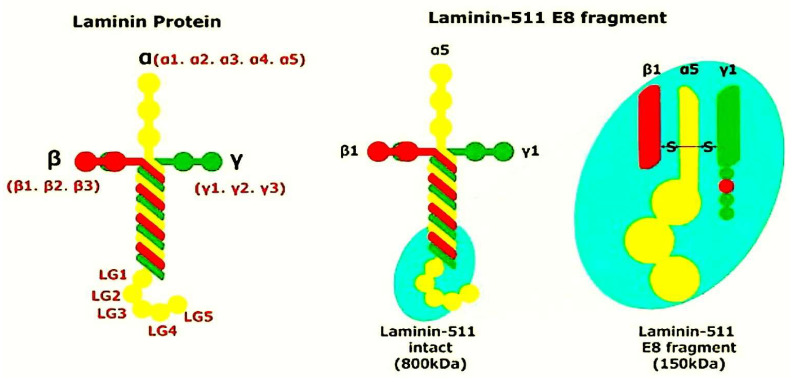
The following illustration outlines the structure of full-length laminin proteins, (yellow) including laminin-511 (yellow and green) and the laminin-511 E8 isoform (yellow and green extended). Merck Research. Development. Production.

Laminins are high-molecular-weight (HMW, 400–900 kD) heterotrimeric adhesion proteins found in BMs, thin sheets comprising muscle tissue, fat, and Schwann cells. Laminins self-polymerize to form a cell, and associated networks play a key role in the development and functionality of BMs. Laminins are found in many organisms. They are composed of an a, a b, and a c chain, each of which is encoded by a distinct gene. Research has revealed that five a, three b, and three c chains have been identified in vertebrates, and that these chains can combine to form over 16 distinct isoforms Figure 5 and Table 1.

As illustrated in Table 2, extracellular matrix lamina substrate is produced through the application of genetic engineering techniques.

Human pluripotent stem cells (hPSCs) express integrin α6β1, enabling their stable, feeder-free growth on laminin-511-coated vessels. However, laminin-511′s large molecular weight and heterotrimeric structure hinder large-scale production. Professor Kiyotoshi Sekiguchi’s research group (Matrixome, Inc.) has achieved a breakthrough in stem cell research. They generated a large-scale recombinant E8 fragment of laminin-511 that maintains full integrin-binding functionality. Laminin Substrates offer numerous advantages over conventional coating methodologies [332,333].

Laminins are named based on their chain composition. For example, laminin-111 is composed of chains α1, β1, and γ1. Chain was the first laminin isoform to be discovered, over 40 years ago. Use of laminin-111 is recommended. Research into this particular laminin isoform is extensive, but its range of expression in adult humans is limited. In summary, laminins are produced in a way that is specific to the type of human tissue, and BMs include at a minimum one laminin isoform [127,334,335,336,337,338,339,340,341,342]. Investigations into the laminin structures have provided valuable insights. All laminin chains exhibit a shared domain structure, comprising globular and rod-like regions. Please find below the details for the α1, α2, and α5. Figure 5 Chains can be assembled into a cross shape. It has three short and one long arm. Laminins can also form T-, Y-, or I-shaped structures. Figure 5 The three short branches of the protein consist of disulfide-rich domains and the N-terminal globular regions (LN domain). These globular structures are present in the main laminin polypeptides and are essential for the polymerization process. Laminin isoforms lacking the N-terminal globular domains (α4, α3A, and γ2 chains) are unable to polymerize, but can still be integrated into BMs, likely through their association with other BM constituents. Laminin molecules are composed of three chains, arranged into a helical coil that ends with a globular C-terminal domain (LG) on the α-chain. The attachment of laminins to the cell surface involves the interaction of their LG domains with various cell surface receptors, including integrins and α-dystroglycan.

The structural characteristics of integrin- and dystroglycan-binding segments of laminins have been thoroughly defined. Laminins have been shown to be involved in complex interrelationships with a range of BM proteins. Laminins are key components in the formation of suprastructural networks that are indispensable for embryonic development and the maintenance of adult organs and systemic functions [96,127,334,335,336,337,338,339,340,341,342]. Mice lacking laminin A1 chain viability have defects in the Reichert’s membrane, an extraembryonic BM present in rats and mice. Absent laminin A1 during foetal development can result in retinal abnormalities, behavioural issues, and aberrant cerebellum development. Research indicates that the absence of laminin A2 chain is responsible for a severe form of muscular dystrophy and can result in death. A deficiency of laminin A3 chain has been observed to cause skin blistering after birth, often leading to death within a few days. Research has indicated that mice lacking the laminin A4 chain exhibit defects in various organs. On the other hand, it has been established that laminin A5 chain is essential for organ formation [96,334,335]. All laminin subgroups have been linked to human pathologies. Mutations in LAMA2, LAMA3, LAMB3, LAMC2, and LAMB2 cause severe congenital disorders, including congenital muscular dystrophy, junctional epidermolysis bullosa, and Pierson syndrome [343,344,345,346]. An array of distinctive cerebral traits are associated with mutations in LAMA1, LAMA2, LAMB1, LAMC1 and LAMC3 [347,348,349,350,351,352,353]. Research has identified a link between mutations in LAMA4 and the development of dilated cardiomyopathy [354,355]. Furthermore, recent studies have highlighted the potential implications of LAMA5 mutations in a range of medical conditions [356,357,358,359,360,361].

Laminins play a crucial role in maintaining BM integrity, facilitating early embryonic development, and supporting organogenesis. They are also essential for preserving the health and survival of numerous tissues. Research has shown that recombinantly expressed laminins are useful tools for developing cell differentiation protocols that are xenogeneic-free and well-defined [362,363]. As illustrated in Table 3, the human laminin genes are located on specific chromosomes, and the corresponding polypeptides, genetic disorders, and OMIM phenotype entries are also delineated.

## 5. Integrins: Mediators of Adhesion and Signalling Between Cells and ECM

### 5.1. Integrins, ECM, and Ligands: A Comprehensive Exploration

Integrins are transmembrane cell adhesion proteins that link the extracellular matrix and the cell cytoskeleton. They are vital to intracellular signalling pathways and their interaction with ECM proteins. Integrins form heterodimers between the alpha (α) and beta (β) subunits, with at least twenty-four unique combinations documented. Keciagia et al. [364] found that integrin subunit a-subunits comprise eighteen types and integrin subunit b-subunits eight types. Alpha- and beta-subunits are type I transmembrane proteins (TMEMs) with a large extracellular domain, a single transmembrane domain, and a short cytoplasmic region [364,365,366]. Harburger et al. [367] found that some subunits are present in greater quantities within a number of different heterodimers. For example, β1 is present in 12 heterodimers and av is present in 5. The extracellular region, notably the aI domain, is responsible for the provision of ligand specificity with ECM macromolecules or counter receptors located on adjacent cells.

**Figure 6:**
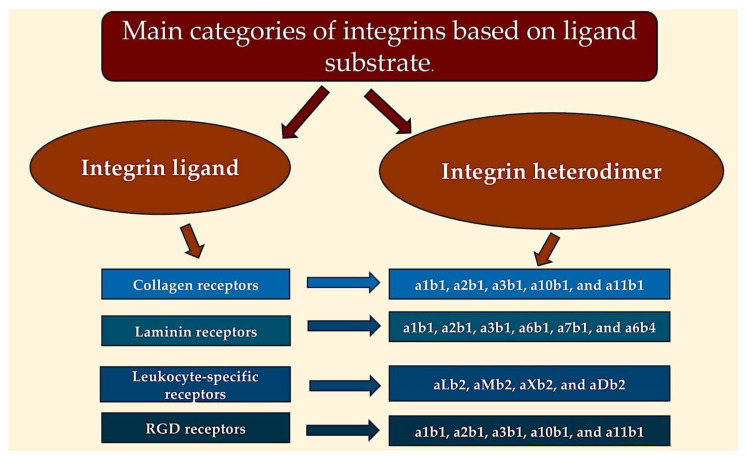
As illustrated in figure, there exists a range of proteins that have the capability of binding to differing integrin heterodimers.

The classification system divides the categories into four groups: collagen receptors, laminin receptors, leukocyte-specific receptors, and the arginine-glycine-aspartic acid (RGD) motif. Talin, a protein that facilitates attachment between a cell and the underlying matrix, is involved in “outside-in signalling.” Wang et al. reveal that talin performs a novel function in linking integrin adhesions with cell cycle progression. Talin-deficient cells exhibited defects in proliferation. Restoring talin’s rod portion, but not its head region, rescued cell cycle progression. Talin employs its C-terminal rod domain to recruit and activate focal adhesion proteins, indispensable for proliferation [365] (Figure 6).

The interaction between extracellular matrix ligands and integrins can be categorised by binding to either both subunits or a specific domain of the α-subunit. In haematopoietic cells, different combinations of α-subunits and β2 integrins provide ligand specificity to integrin heterodimers. RGD-binding integrins have an RGD ligand that binds to the interface between the α and β subunits. The interaction between extracellular matrix ligands and integrins can be categorised by binding to either both subunits or a specific domain of the α-subunit. In haematopoietic cells, different combinations of α-subunits and β2 integrins provide ligand specificity to integrin heterodimers. RGD-binding integrins have an RGD ligand that binds to an interface between the α and β subunits [367,368,369,370].

Fibronectin, VCAM-1, and MAdCAM-1 contain an LDV motif and bind to several integrins, including α4β1, α4β7, and α9β1 [370]. Osteopontin binds to α4β1, α4β7, and α9β1 via the SVVYGLR peptide motif [371,372,373,374,375]. The A-domain within the A subunit facilitates the binding of ligands to numerous b-subunit families. β2 family-specific ligand sites are similar to the LDV motif. β2 uses glutamate instead of aspartate for cation coordination (Table 2) [376]. The A-domain (found in the α1, α2, α10, and α11 subunits) has been observed to form hexamer-dimer structures with the β1 subunit, creating laminin- and collagen-binding families [370]. The α2A domain has been observed to interact with collagen via the GFOGER sequence ([377,378]). Non-αA domain-containing integrins, such as α3β1, α6β1, α7β1, and α6b4, exhibit a high degree of affinity for laminin binding proteins. As presented in Table 3, there is a reported interaction between proteins and various integrin heterodimers.

### 5.2. The Process of Integrin Activation, Its Functions in Physiological Contexts and Its Impact on the Disease

Research on integrin regulation began with blood cells [379,380]. While studies have mostly focused on platelets and leukocytes, integrins are present in many cell types and play a significant role in angiogenesis, cell migration, and ECM remodelling. As outlined by Tadokoro et al. [381], integrin activation involves talin binding to the cytoplasmic tail of the β1 subunit. Talin binding changes the structure of both subunits by separating the cytoplasmic region and extending the extracellular region. This higher affinity with the ligands results from these changes. Additional factors contribute to the activation process and facilitate the assembly of diverse adhesive complexes following the initial interaction between talin and the cytoplasmic domain [382]. Kindlin is a key player in inside-out integrin signalling, influencing ECM interactions and cell spreading through the activation of the beta-subunit cytoplasmic tail or the recruitment of focal adhesion. Paxillin, an activator of RHO GTPase, is a key molecule in this process [382,383,384]. Bottcher et al. state that RAC1 polymerizes actin using the Arp2/3 complex, facilitating cell spreading. During adhesion maturation, talin-induced integrin is maintained by tensin-1 and tensin-3 binding to the β1 subunit [385]. The precise integrin–tensin mechanism is unclear, but talin adhesion transitions to tensin adherence during cell-to-cell interactions due to proximity of their binding sites on the b1 subunit [386,387,388,389]. Integrins play a key role in cell-ECM interactions, influencing downstream signalling pathways. These pathways regulate processes such as cell death or survival, cytoskeleton dynamics, and cell migration, contributing to tissue integrity [390].

Molecules in the extra-cellular matrix regulate embryonic development and cancer progression, as evidenced by their impact on structure and biochemical signals [391]. Integrins in this network control cell migration and influence the interaction between substrate-bound ligands within the ECM environment. Two factors must be considered: the presence of diffusible ligands and the rigidity of the ECM. Integrin-mediated signalling pathways regulate the velocity of migratory cells in response to the rigidity of the ECM [392]. Fibronectin and collagen are the most abundant ECM proteins. These proteins are found in the developing organism. Both proteins are essential for the assembly of the ECM, since the process of fibrillar collagen secretion is dependent on the fibronectin protein complex [393]. Fibronectin-binding integrins are indispensable for the formation of fibronectin networks. Integrin heterodimers, such as a5b1, transduce forces to fibronectin, unveiling cryptic binding sites that are essential for protein polymerization [394]. CAFs accumulate excess ECM components, particularly collagen, in the context of a malignant lesion [395]. The increased deposition of collagen is triggered by the activation of integrin signalling pathways, including FAK and Yap/Taz.

Totaro et al. [396] demonstrated that pathways drive the progression of the disease. Collagen promotes cancer progression, and recent findings identified a connection between fibronectin deposition and prostate cancer invasion. The remodelling of fibronectin via traction forces and the interaction between integrin α5 and β1 generate fibres that promote cell migration [397]. In breast malignancies, the TWIST1 transcription factor increases the expression of the β1 subunit, contributing to cancer invasion. TWIST1 is an EMT transcription factor associated with poor-prognosis cases of metastatic cancers [398]. Evidence shows integrins’ involvement in complex interactions with GF receptors and syndecans [399]. Inhibition of IGFR signal transduction in MCF-7 breast cancer cells has been shown to decrease cell adhesion to fibronectin and laminin. This is due to reduced α5β1, αmβ3, αmβ5, and αmβ6 integrin expression at the cell surface. Previous studies have documented syndecan-4 endocytosis [400,401].

### 5.3. Fibrillin Dysregulation in Bicuspid Aortic Valve Disease

The ECM is very useful. It provides support for the structure and environment, which are necessary for normal tissue development and health. ECM proteins control cell changes and form the valves during heart valve development. Proteins help form the structures that hold embryonic cells together and regulate tissue formation in developing aortic valves. The change in cushion mesenchymal cells into mature valve cells is related to the production of the protein microfibrillar, with proteins fibrillin and fibulin also involved [402]. Some patients with BAV have a lack of microfibrillar proteins in the aortic matrix. This can cause problems with the formation of the aortic cusps. Early data suggest that microfibrillar proteins may be missing in adults with BAV disease, which can cause problems with the structure of the aortic root [403]. So far, there have been no problems with the genes that control the matrix elements in patients with BAVs. While the fibrillin-1 gene may be structurally intact, the elements that regulate protein production levels may be compromised. Transcription elements have been demonstrated to be significant in many congenital cardiac pathologies. Another important factor is the gene that governs the synthesis of nitric oxide, a chemical that is important for the cardiovascular system [404]. Mice with this gene problem often have a high risk of a heart problem called bicuspid aortic valve (BAV) [405]. While there is probably a mixed set of causes leading to different clinical symptoms, scientists are sure to find the molecules and cells involved in this common disorder using new methods in molecular biology.

**Figure 7:**
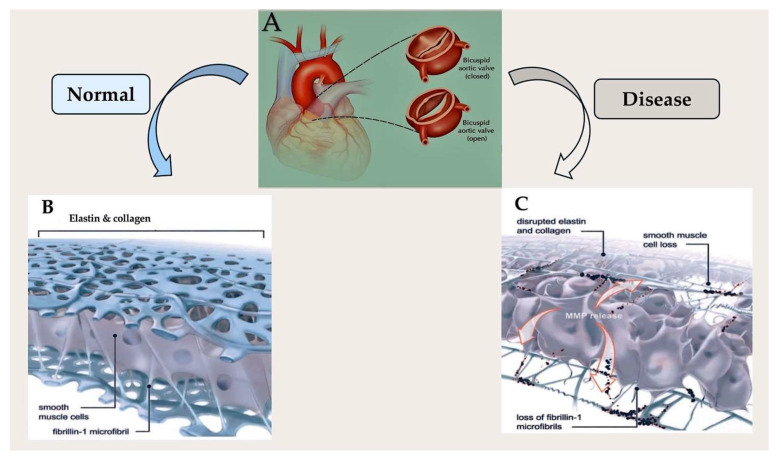
(**A**) Aortic valves exhibiting bicuspid morphology are indicative of aortic valve disease. The aortic media has elastic laminae that provide support and elasticity to the aorta. This is normal for the tricuspid valve (blue arrow). Valve patients (**B**) have fibrillin-1 microfibrils that attach smooth muscle. Cells are joined to the parts around them that are made of elastin and collagen. In patients with BAV (**C**) the body does not make enough of the microfibrillar elements that are needed. Smooth muscle cells detach, releasing enzymes called MMPs, which disrupt the matrix. (grey arrow) This leads to cell death and a loss of structural support and elasticity [406,407,408,409,410,412,413,414,415,416,417,418].

BAV is linked to the rapid deterioration of the aortic media, showing that BAV disease is a continuous pathological process, not a single event. Investigations have found problems in the brain [406,407,408,409,410,411]. In patients with BAV, the inner lining of the aorta can break down and the muscle cells can shrink, indicating gradual weakening. The lesions in these two cases are similar to those seen in fibrillin-1-deficient aortas [412,413] and in patients with Marfan syndrome [414]. A loss of fibrillin-1 microfibrils can cause smooth muscle cells to separate from the stuff that makes up the middle of the body [412,413,414]. This can lead to cell death and tissue breakdown. Matrix metalloproteinases (MMPs) are enzymes that break down body tissues. Research shows that these enzymes may play a role in the development of atherosclerotic aortic aneurysms [415]. When fibrillin-1 is absent, MMPs become active, causing the aorta’s structural support to break down. This can lead to widening, bulges, and potentially rupture [410,411,416,417,418]. Initial findings indicate that MMP may be present in the aorta of patients with BAV. Understanding how the body responds to aortic problems in these patients may reveal the genes causing them and how to make them less dangerous Figure 7.

## 6. Remodelling of the ECM and Its Dynamic Nature

Tissue growth, repair, renewal, and internal equilibrium require the dynamic character of the ECM, a process termed “remodelling” [5,14]. As the literature shows, any modifications to this procedure that is well-balanced can result in pathogenesis [6,106,112,113]. The HPSE enzyme cleaves the side chains of Hep and HS of PGs during the process of shedding. HPSE can be detected within the ECM under appropriate stimulation conditions [419,420]. HPSE, when used with HYALs, which can degrade HA, has been shown to modify GAGs in ECM, altering their structure and function. HPSE’s role is crucial in determining the fate of Hep and HS interacting partners, like GFs, cytokines, and enzymes, which are vital in remodelling the ECM. HPSE triggers signalling cascades, including Akt, ERK, p38, and Src, through its engagement with TMEMs. HPSE regulates biological processes like cell motility, angiogenesis, inflammation, exosome biogenesis, and cellular autophagy [421]. Proteinases are enzymes that regulate cellular processes and rearrange ECM components. The metzincin superfamily comprises metalloproteinases (MMPs) and adamalysins. The group under discussion consists of disintegrin and metalloproteinases (ADAMs) and ADAMTSs [2,422,423]. MT-MMPs and most ADAMs are TMEMs, suggesting the potential to manipulate the local microenvironment of BMs. Researchers posit that ADAMs are sheddases that interact with cytokines, growth factors (GFs), and their receptors, regulating cell migration, adhesion, and fate determination [423,424,425].

### 6.1. ADAMTSs, ADAMs and MMPs

Matrix metalloproteinases (MMPs) are a subfamily of proteolytic enzymes that fall into the metzincin clan along with ADAMs (a disintegrin-like proteins) and ADAMTSs (a disintegrin and metalloprotease domains containing proteins). These cells are key to maintaining ECM homeostasis and regulating autocrine and paracrine signalling. MMPs and ADAMTSs are secreted enzymes, but certain MMPs and several ADAMTSs are located on the cell surface or within the pericellular environment [426,427]. ADAMs (a disintegrin-like and metalloproteinases) are a subfamily of TM (Toll-like) metalloproteinases that act on membrane-localised substrates.

Enzymes like matrix metalloproteinases, ADAMTSs, and ADAMs contain a conserved catalytic region and ancillary segments that modulate substrate affinity and enzyme compartmentalization. The proteolytic function of metzincins depends on an active site Zn2+ ion, strategically coordinated by three histidine residues. This coordination occurs within a well-conserved HEXXHXXGXX(H/D) motif, a hallmark of protein tertiary structural elements [428]. Enzymes like MMPs, ADAMTSs, and ADAMs have a conserved catalytic region and ancillary segments that modulate substrate affinity and enzyme compartmentalization. The nomenclature indicates that metzincins’ proteolytic function depends on an active site Zn^2+^ ion, coordinated by three histidine residues. This coordination follows a conserved HEXXHXXGXX(H/D) pattern, a feature of protein tertiary structure [428]. The glutamic acid residue in this motif activates a Zn^2+^-bound H_2_O molecule, creating a nucleophile that cleaves substrate peptide molecules. A methionine residue is also present in the downstream region of the catalytic module in metzincins. This residue forms the Met-turn, a structural element that determines the substrate-binding pocket within the catalytic domain [429].

### 6.2. Insight on Physiological and Pathological Functions of Matrix Metalloproteinases

The terms “matrix metalloproteinases” (MMPs) were first derived from their substrates and functions. These functions include ECM molecule degradation, contributing to tissue homeostasis and remodelling. MMPs influence many factors. The categorization of MMPs is based on their substrates and homology. Substrates are divided into six groups: collagenases, stromely-sins, matrilysins, gelatinases, furin-activated MMPs, and other MMPs. The molecular mechanisms underlying the effects of these enzymes are monitored at the transcriptional level. This includes activating the inactive zymogens and interacting with tissue inhibitors (TIMPs). MMP-1 activates JNK and ERK molecules, which trigger the differentiation of bone mesenchymal stem cells [430,431].

The prototype of the matrix metalloproteinase family, designated MMP-1, was discovered to be the enzyme responsible for the degradation of type I collagen. This process occurs during tadpole tail resorption, a feature of metamorphosis. In addition to the cysteine protease cathepsin K, the matrix metalloproteinases (MMP-1, MMP-2, MMP-8, MMP-13, and MMP-14) are the only mammalian enzymes that can cleave native triple-helical collagen [426]. Additional MMP family members were found to be crucial for matrix metabolism, specifically the degradation of structural proteins during wound healing and angiogenesis [432]. Subsequent research has revealed the subtle roles of these enzymes in protein processing. Their proteolytic activity activates or deactivates bioactive substrates, including GFs, cytokines, cell surface receptors, and adhesion molecules. MMPs regulate a variety of biological processes, such as inflammation and immune function. Tissue repair and differentiation are complex phenomena.

MMPs have pathological functions in various diseases, including cancer, cardiovascular disease, osteoarthritis, and emphysema [433,434,435,436]. MMPs play a pivotal role in processes such as cancer invasion and metastasis because they can degrade basement membranes and other components of the extra-cellular matrix. They have been shown to stimulate tumour growth and survival [437,438]. Clinical trials of matrix metalloproteinase inhibitors for cancer treatment met with limited success due to the inhibitors’ low specificity, which led to unintended inhibition of important metalloproteinases. This led to unwanted side effects [438,439]. A deeper understanding of MMP biology has led to the development of more selective and target-specific inhibitors that may work better in clinical settings [440,441,442,443]. HPSE-1 is implicated in various pathological conditions due to its role in degrading HSPG and signalling mechanisms. Evidence suggests a link between HPSE-1 expression and tumour development and inflammatory responses [444,445,446,447,448,449]. Table 4 shows degenerative diseases as well.

### 6.3. ADAMTSs and ADAMs

#### Physiological and Pathological Functions

It has been demonstrated that the 19 mammalian ADAMTSs are important for the maintenance of the ECM in adult and embryonic tissues [450]. Many of the ADAMTSs have been identified, but much remains to be learned about the functions and substrate repertoires of this structurally complex group of enzymes. These enzymes are specific to certain substrates, affecting collagen maturation, the cleavage of PG, blood coagulation and angiogenesis [451,452]. The proteinases ADAMTS-2, ADAMTS-3 and ADAMTS-14 cleave the aminopropeptide of fibrillar procollagens, promoting collagen maturation and ECM assembly [453]. Mutations in ADAMTS2 cause a rare form of Ehlers-Danlos syndrome, characterised by weakening of connective tissue and skin fragility. Additional roles in TGFb signalling are emerging [454]. Research has shown that ADAMTS-7 and -12 have the ability to cleave COMP, which contributes to matrix degradation in arthritis [455]. Further research has recently revealed new cardiovascular precursors of ADAMTS-7, along with the preferential inhibition of this process by TIMP-4, which is selectively expressed in cardiovascular tissues [456]. ADAMTS-13 plays a key role in regulating hemostasis by cleaving vWF multimers into smaller fragments, thereby controlling their interaction with platelets. Reduced ADAMTS-13 activity, caused by mutations or autoantibodies, can lead to thrombotic thrombocytopenic purpura [457]. Cleavage is regulated by shear stress-induced changes in both vWF and ADAMTS-13 [458].

ADAMs are metalloproteinases involved in development, cell migration and interaction with TIMP-2 and ECM PGs. Tissue inhibitor of metalloproteinases are widely expressed, with the exception of TIMP-4, which is found primarily in the brain, heart and adipocytes. TIMP expression is dysregulated in pathologies such as cancer, osteoarthritis and fibrosis [459].

### 6.4. Heparanases

#### Physiological and Pathological Functions

HPSE-1 expression is strictly regulated in healthy tissues to prevent uncontrolled degradation of HSPGs. In most tissues, its expression is limited by epigenetic regulation of the HPSE-1 promoter [460] and by the activity of the transcription factor p53 [461]. Consequently, HPSE is expressed in keratinocytes, trophoblasts, platelets and some immune cells [462]. During early mouse gestation, HPSE-1 increases in the uterus, contributing to tissue remodelling and the release of GFs necessary for blastocyst implantation and embryo development [463]. HPSE-1 is required for wound healing as it stimulates tissue repair [464]. It also facilitates the interaction of leukocytes with subendothelial BM and their extravasation as well as blood clotting [462]. HPSE-1’s degradative function and signalling mechanisms make it a key player in various pathological conditions. Its expression in tumours, inflammatory and degenerative diseases is shown in Table 4. Mutated p53, oestrogen, ROS, hypoxia, inflammatory cytokines, hyperglycaemia and albuminuria are some of the factors causing its over-expression. HPSE-1 is a key player in almost all human cancers [465]. It has been shown to be involved in tumour initiation, angiogenesis, growth, metastasis and chemoresistance [466,467].

## 7. Future Direction

Despite the strong relationship between many matrix components and disease progression, the ECM field, while growing, has been overlooked in new treatments. The data here could support further research on the role of ECM. It could also help doctors and patients discuss benefits and expectations after a better understanding of ECM. The ECM research collective should study the molecular mechanisms that regulate matrix-based pathologies to develop strategies for future pharmacological targeting, disease diagnosis, and prognostication. BAV advances in imaging, genetics and risk assessments should be used to understand bicuspid aortic valve disease. The Beta Blockers and Angiotensin Receptor Blockers in Bicuspid Aortic Valve Disease Aortopathy (BAV) study (NCT01202721) is an ongoing trial by the Canadian Network and Centre for Trials. The study aims to evaluate the efficacy of atenolol and telmisartan in slowing the progression of bicuspid aortopathy. A trial of medical versus surgical management in patients with moderately dilated ascending aorta (45 to 50 mm) would be informative. Future studies should focus on selecting subgroups of patients most likely to benefit from earlier intervention or watchful waiting [468].

## 8. Conclusions

**Figure 8:**
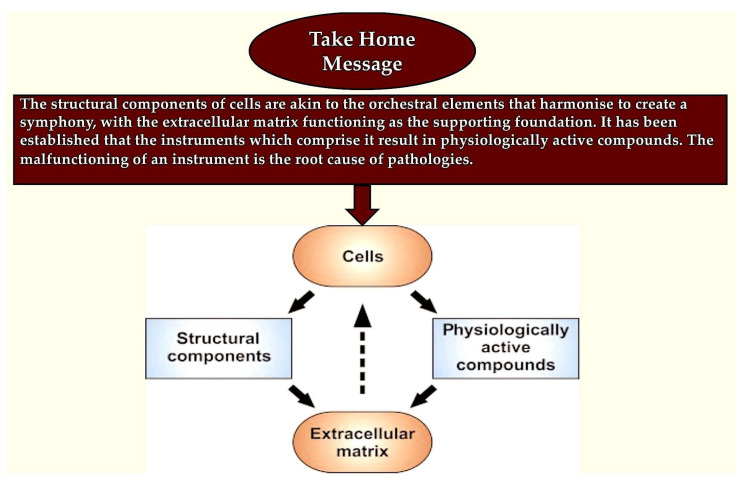
The core finding regarding the pathophysiology of ECM encompasses structural components and physiologically active compounds, highlighting a comprehensive understanding of its multifaceted nature.

The evidence definitively shows that a genetic basis exists for BAV. First-degree relatives of patients suffering from BAV disease are highly likely to suffer from aortopathy, as the following observations definitively prove [469]. The inheritance of this condition has been observed to follow autosomal dominant, X-linked and familial modes [1,4,470]. Furthermore, it has been hypothesised that abnormal migration of neural-crest cells is a shared pathway that leads to the development of bicuspid aortic valves and aortopathies [470,471]. To understand bicuspid aortopathy better, we need state-of-the-art risk assessments and genetic analysis of ECM. This review provides a comprehensive account of ECM composition and function, with robust evidence from extensive bibliography. The study design is based on a clinical case of BAV. It aims to investigate the ECM. Recent research has illuminated progress in the field of ECM macromolecules and network interrelationships. The primary function of the ECM is to regulate physiological processes. The ECM is vital for tissue integrity, cell signalling and gene expression. While we have much knowledge, learning is still vital. Belief is crucial to understanding the ECM. This review describes and synthesises this complex field, explaining its key components and functions. ECM complexes contain many macromolecules, including collagen and its receptors, elastin, laminins, several PGs, and hyaluronan, which is associated with its receptor, CD44. Matrix-degrading enzymes impact tissue remodelling in physiological and pathological statesin Figure 8.

## Figures and Tables

**Table 1:** Key points.

The bicuspid aortic valve (BAV) is the most common congenital cardiac malformation. Pathophysiology of this disease are not well understood The extracellular matrix (ECM) is integral to the provision of structural integrity and environmental signals that are essential for the regulation of normal tissue development and homeostasis. Patients with BAV may have a deficiency of microfibrillar proteins in the aortic matrix. Disruptions in the formation of the aortic cusps may result in a bicuspid valve and a weakened aortic root due to inadequate production of fibrillin-1 during valvulogenesis. Investigating genetic, biomolecular, and histopathological processes that lead to changes in the structure of the ECM is useful for understanding, diagnosing, and treating related diseases.

**Table 2:** Extracellular matrix lamina substrate, produced through the application of genetic engineering techniques.

Laminin Isoform	111	211	121	221	332	311	321	411	421	511	521
α	α1	α2	α1	α2	α3	α3	α3	α4	α4	α5	α5
β	β1	β1	β2	β2	β3	β1	β2	β1	β2	β1	β2
γ	γ1	γ1	γ1	γ1	γ2	γ1	γ1	γ1	γ1	γ1	γ1

**Table 3:** Proteins interacting with various integrin heterodimers.

Binding Protein	Integrin Heterodimer
Fibronectin	α v β 3, α v β 6, aIIb β 3, α v β 1, α 5 β 1, α 8 β 1, α 4 β 1, α 4 β 7
vWF	α v β 3, α IIb β 3
Vitronectin	α β 3, α v β 5, α 8 β 1, α IIb β 3
Fibrillin	α v β 3
Fibrinogen	α v β 3, α IIb β 3, α X β 2, α M β 2
Factor X	α M β 2
Developmental endothelial locus-1	α β 3, α v β 5
Inactivated complement component C3b	α X β 2, α M β 2
TN	α v β 3, α 8 β 1, α 9 β 1
Platelet endothelial cell adhesion molecule 1	α v β 3
E-cadherin	α E β 7
Latency associated peptide transforming GF	α v β 1, α v β 8, α v β 6, α v β 3
Mucosal addressin cell adhesion molecule 1	α 4 β 7, α 4 β 1
Bone sialoprotein	α v β 3, α v β 5
Thrombospondin	α 3 β 1, α 2 β 1, α 4 β 1, α v β 3, α 3 β 1, α IIb β 3
Laminin	α 3 β 1, α 6 β 1, α 6 β 4, α 7 β 1, α 1 β 1, α 2 β 1, α 10 β 1
Collagen	α 1 β 1, α 5 β 1, α 10 β 1, α 11 β 1, α X β 2
OPN	α 4 β 7, α 4 β 1, α 9 β 1, α 8 β 1, α 5 β 1, α v β 1, α v β 6, α v β 3, α v β 75

**Table 4:** The role of heparanase in disease.

Pathological condition ♦ Cancer signaling such as FGF2, TGFb, HGF, VEGF and EGF HPSE inhibits apoptosis viaHS-mediated signaling Autophagy regulation Inducing angiogenesis Activating invasion, metastasis, EMT and EC degradation Involving in exosome formation ♦ Inflammation ♦ Diabetes ♦ Coagulation dysfunctions ♦ Amyloid disease ♦ Kidney disease ♦ Organ fibrosis ♦ Pancreatitis ♦ Viral Infection

## Data Availability

Not applicable.

## Abbreviations

ADAM	a disintegrin and metalloproteinase
ADAMTS	a disintegrin and metalloproteinase with thrombospondin motifs
AML	acute myeloid leukaemia
BAV	bicuspid aortic valve
BM	basement membrane
CAF	cancer-associated fibroblast;
CD44	cluster of differentiation 44
CNS	central nervous system
CR-CSCs	colorectal cancer stem cells
CS	chondroitin sulphate
DDRs	discoidin domain receptors
DES	desmosine
DS	dermatan sulphate
EBP	elastin-binding protein
ECM	extracellular matrix
EDPs	elastin-derived peptides
EGFR	epidermal growth factor receptor
EMILINs	elastin microfibril interfacers
EMT	epithelial-to-mesenchymal transition
ERC	elastin receptor complex
ERM	ezrin–radixin–moesin
FACITs	fibril-associated collagens with interrupted triple helices
FGFR	fibroblast growth factor receptor
GAG	glycosaminoglycan
GFs	growth factors
GPC	glypican
GPI	glycosylphosphatidylinositol
HA	hyaluronan;
HAS	hyaluronan synthase
HB-EGF	heparin-binding EGF
Hep	heparin
Hh	Hedgehog
HPSE	heparanase
HS	heparan sulphate
Hyal	hyaluronidase
IGFIR	insulin-like growth factor receptor I
LacCer	lactosylceramide
LOX	lysyl oxidase
LRP	lipoprotein receptor-related protein
MACITs	membrane-associated collagens with interrupted triple helices
MET	mesenchymal-to-epithelial transition
MMPs	matrix metalloproteinases
Neu-1	neuraminidase-1
OPN	osteopontin
PEGF	pigment epithelium-derived factor
PG	proteoglycan
RHAMM	receptor for hyaluronan-mediated motility
RIP	regulated intramembrane proteolysis
ROS	reactive oxygen species
RTK	receptor tyrosine kinase
SARS-CoV-2	Severe acute respiratory syndrome coronavirus 2
SLRPs	small leucine-rich proteoglycans
SRGN	serglycin
TE	tropoelastin
TIMP	tissue inhibitor of metalloproteinases
TLR	toll-like receptor
TMEM	transmembrane protein
TN	tenascin
TSP	thrombospondin
VEGF	vascular endothelial growth factor
vWF	vonWillebrand factor.
